# The first record of the anopsobiid genus *Shikokuobius* Shinohara, 1982 in continental Asia, with the description of a new species from the Altais, southwestern Siberia, Russia (Chilopoda, Lithobiomorpha, Anopsobiidae)

**DOI:** 10.3897/zookeys.793.29221

**Published:** 2018-10-29

**Authors:** Gyulli Sh. Farzalieva, Pavel S. Nefediev

**Affiliations:** 1 Department of Invertebrate Zoology and Aquatic Ecology, Perm State University, Bukireva 15, Perm 614990, Russia Perm State University Perm Russia; 2 Department of Ecology, Biochemistry and Biotechnology, Altai State University, Lenina 61, Barnaul 656049, Russia Altai State University Barnaul Russia; 3 Biological Institute, Tomsk State University, Biological Institute, Lenina 36, Tomsk 634050, Russia Tomsk State University Tomsk Russia

**Keywords:** Centipede, taxonomy, new species, Altai Mountains, Siberia, Russia

## Abstract

A new lithobiomorph species, *Shikokuobiusaltaicus***sp. n.**, is described from the Altai Mountains in southwestern Siberia, Russia. This is the first record of the genus *Shikokuobius* Shinohara, 1982 in continental Asia, all previous reports being from Japan. The distribution of *Shikokuobius* is mapped.

## Introduction

The family Anopsobiidae Verhoeff, 1907 is currently known to be represented in the Northern Hemisphere by one small and eight monotypic genera: *Yobius* Chamberlin, 1945 (Utah, USA), *Hedinobius* Verhoeff, 1934 (Tien Shan, western China), *Rhodobius* Silvestri, 1932 (Europe), *Anopsobiella* Attems, 1938 (Vietnam), *Shikokuobius* Shinohara, 1982 (Japan), *Ghilaroviella* Zalesskaja, 1975 (Tajikistan), *Dzhungaria* Farzalieva, Zalesskaja et Edgecombe, 2004 (eastern Kazakhstan), *Speleopsobius* Shear, 2018 (southern Idaho, USA), as well as *Buethobius* Chamberlin, 1911, with 5 species from the USA (Silvestri 1909, Attems 1938, Zalesskaja 1975, Shinohara 1982, Farzalieva et al. 2004, Zapparoli and Edgecombe 2011, Shear 2018).

A collection of lithobiomorph centipedes from the Republic of Altai, Russia, deposited in the Zoological Museum of the Lomonosov Moscow State University, has yielded a single male of a new anopsobiid species of *Shikokuobius*. Four additional specimens of that new species have also been freshly collected from the Altai Province, Russia.

*Shikokuobiusaltaicus* sp. n. is very similar to *S.japonicus* (Murakami, 1967) from Japan (Sakuragi, Honshu; Nakameguro and Shirogane; the DNA voucher specimen of Edgecombe and Giribet (2003, 2004), Shizen-Kyoiku-en, Meguro-ku, all in Tokyo). The present paper describes the new species and refines the distribution of the genus *Shikokuobius*.

## Material and methods

The material used in the present study was collected by S.I. Golovatch (Moscow, Russia) in the environs of Lake Teletskoye, Republic of Altai and by T.M. Krugova (Barnaul, Russia) with her team of volunteers in the Tigirek State Nature Reserve, Altai Province. Both sites are located in the Russian Altais, southwestern Siberia, Russia. Most of the material is currently deposited in the collection of the Zoological Museum of the Lomonosov Moscow State University, Moscow, Russia (ZMUM), partly also shared with the collection of the Perm State University, Perm, Russia (PSU).

The total body length was measured from the fore margin of the cephalic plate to the posterior end of the postpedal tergite. Leg length was measured excluding the length of the claw. Lengths are given as the minimum and maximum values. All measurements are given in millimeters (mm).

The mouthparts, legs and body segments of the new species were cleared in 10% KOH and mounted in permanent slides in sandarac medium (Krasheninnikov 2011) for examination. The specimens were examined and measured using a Meiji EMZ-5 stereo microscope, and stacks of colour images were manually generated using an Olympus OMD EM-10 digital camera with a Panasonic Lumix H-H025 25 mm f/1.7 lens mounted on a Zeiss microscope. Digital images were prepared using Photoshop CS6 image stacking software. The drawings were executed using a Zeiss microscope and a Zeiss drawing tube. The distribution map was composed using QGIS 3.0.

The terminology of the external anatomy follows Bonato et al. (2010).

The following abbreviations are used in the text:

T, TT tergite, tergites;

C, CC coxa, coxae;

t trochanter;

P prefemur;

F femur.

## Results

### Taxonomy

#### Shikokuobius altaicus sp. n.

Taxon classificationAnimaliaLithobiomorphaAnopsobiidae

http://zoobank.org/34A98474-0F2E-41D8-9E7A-A1E84EE30F4C

Figs 1–6 , 7–13 , 14–19 , 20–24 , 25–29 , 30–35 , 36–43 , 45

##### Type material.

Holotype ♂ (ZMUM, Rc 7867): Russia, southwestern Siberia, Altai Province, Krasnoshchiokovo District, near Tigirek village, buffer zone of the Tigirek State Nature Reserve, foot of W slope of Mt. Kozyr, 51°09'26.54"N, 83°00'01.59"E, thicket of *Padusavium*, *Loniceratatarica* and *Viburnumopulus*, 600 m a.s.l., soil sample (30–40 cm deep), 29–30.08.2016, leg. T.M. Krugova, L.Yu. Gruntova, V.V. Zelensky, K.V. Smirnova, A.E. Pupkova, M.N. Terioshkina and R.V. Scherbakova.

##### Paratypes.

1 ♂ (ZMUM, Rc 7868), 1 ♀ (ZMUM, Rc 7869), together with holotype, soil samples (30–40 cm deep and 0–10 cm deep, respectively); 1 ♀ (PSU-612), Russia, southwestern Siberia, Altai Province, Krasnoshchiokovo District, near Tigirek village, Tigirek State Nature Reserve, Khankhara Site, right bank of Khankhara River, upper part of N slope, 51°11'35.36"N, 82°58'48.26"E, *Larixsibirica* forest with *Betulapendula*, 920 m a.s.l., soil sample (20–30 cm deep), 19.08.2016, leg. T.M. Krugova, L.Yu. Gruntova, V.V. Zelensky, K.V. Smirnova, A.E. Pupkova, M.N. Terioshkina and R.V. Scherbakova.

##### Non-type material.

1 ♂ (PSU-536), [Russia, southwestern Siberia, Republic of Altai, Turochak District], near Lake Teletskoye, environs of Artybash village [51.814745N, 87.278742E, ca 450 m a.s.l.], *Betulapendula*, *Abiessibirica* and *Pinussibirica* forest, litter, 4–13.07.1982, leg. S.I. Golovatch.

##### Name.

The specific epithet refers to Altai, the *locus typicus*.

##### Diagnosis.

**Figures 1–6.**
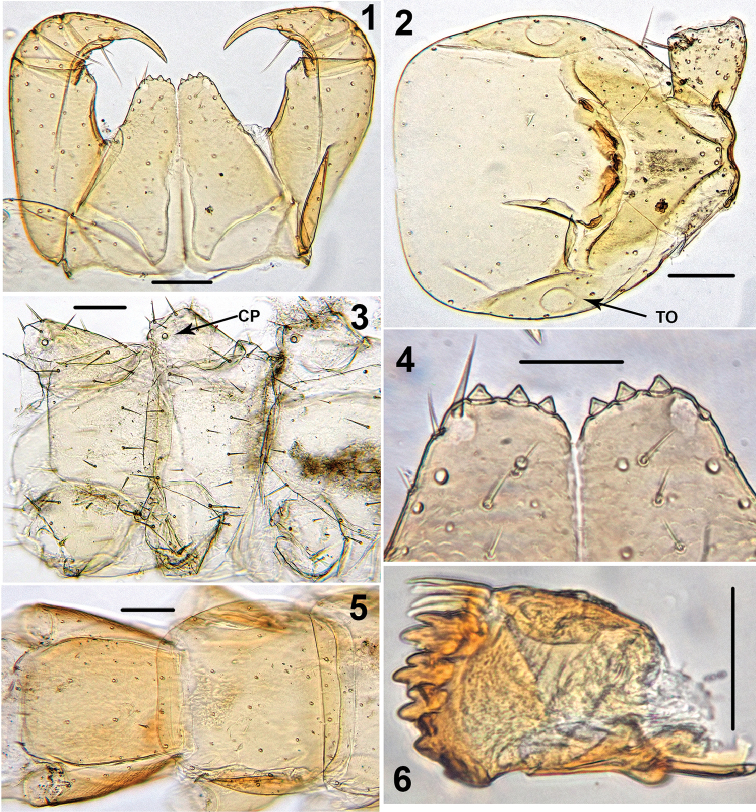
*Shikokuobiusaltaicus* sp. n., male paratype (**1, 3, 6**) and female paratype (**2, 4, 5**). **1** forcipulae, ventral view **2** head, ventral view **3** 12–14 sternites and coxae, ventral view **4** dental margin of forcipular coxosternite **5** 13–16 tergites, dorsal view **6** mandibula, ventrolateral view. Abbreviations: **TO** Tömösváry’s organ, **CO** coxal pore. Scale bars: 0.1 mm (**1–5**), 0.05 mm (**6**).

**Figures 7–13.**
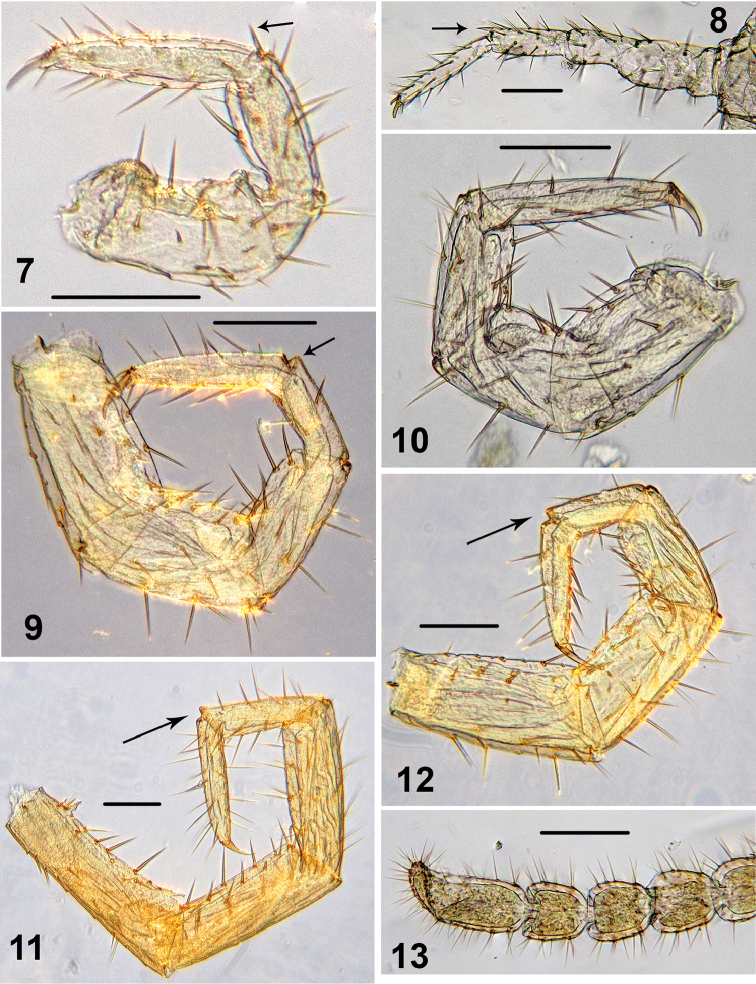
*Shikokuobiusaltaicus* sp. n., male paratype, lateral views. **7** leg 1 **8** leg 3 **9** leg 14 **10** leg 12 **11** leg 15 **12** leg 13 **13** 5 terminal antennomeres. Scale bars: 0.1 mm (**7, 9–13**), 0.5 mm (**8**).

A *Shikokuobius* species with the body 4–6 mm long, the antennae composed of 15–17 antennomeres, commonly 15; coxosternal teeth large, separated from each other by distances less than width at the base of a tooth; P, F and T of 15 leg relatively short and thick, 15 C with a prominent, acute, mesodistal process; the number of coxal pores varying from 1 to 2 on 12–15 CC (formula 1,1,1,1 in the male and 1,1,1,2 in the female); 15 t and 15 P with bifurcate ventral spines (seldom on legs 13 and 14); at least each leg 15 with a bifurcate spine at the distodorsal end of P; 1–10 tibiae with a distal spinose projection (Figs 7–8, 24); 1–12 tarsi clearly unipartite, claws of 1–14 legs with two accessory spines, claw of legs 15 with a single accessory spine; 1^st^ female gonopodal segment with 2+2 coniform spurs and eight long setae, 2^nd^ with four setae, 3^rd^ with a single seta on the external face; terminal claw simple; male gonopod with four segments including terminal filament.

##### Distribution

(Fig. 46). Altai Province and Republic of Altai, southwestern Siberia, Russia.

##### Description.

**Figures 14–19.**
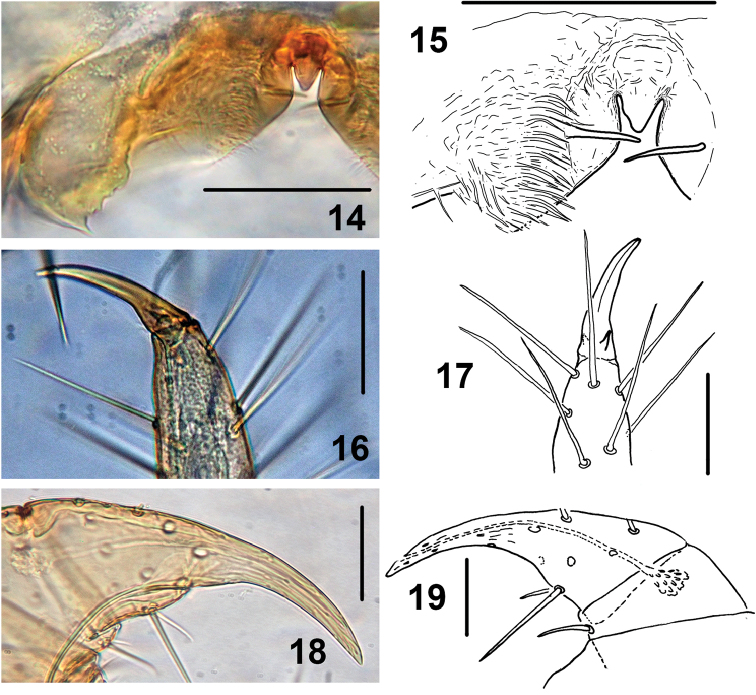
*Shikokuobiusaltaicus* sp. n., male paratype (**14–17)** and female paratypes (**18, 19**). **14, 15** labrum, ventral views **16, 17** apical claw of leg 15, ventrolateral views **18, 19** apical part of forcipular telopodite, ventral views. Scale bar: 0.05 mm.

**Figures 20–24.**
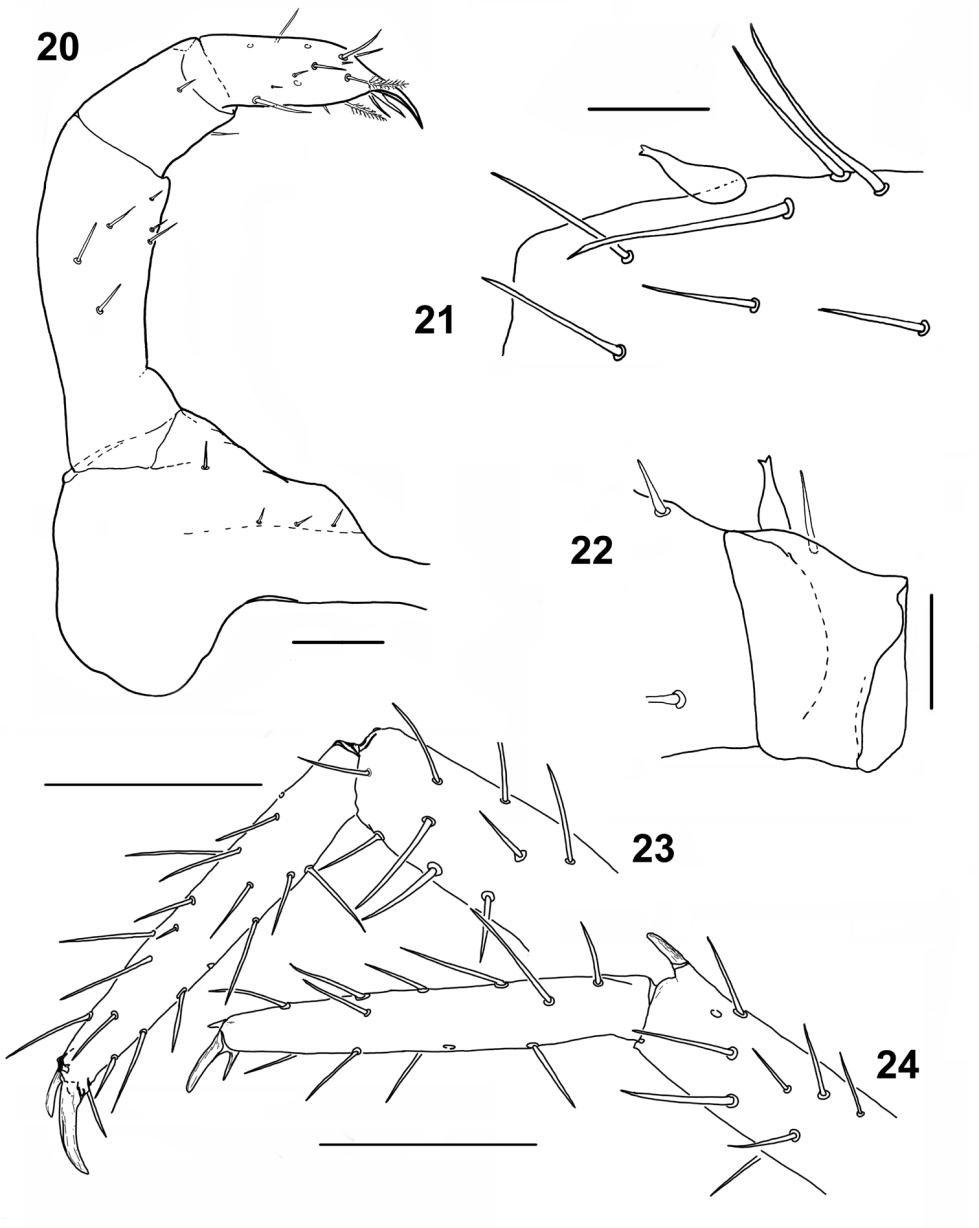
*Shikokuobiusaltaicus* sp. n., male holotype (**20–22**) and male paratype (**23, 24**). **20** left part of second maxilla, ventral view **21** ventral spine on prefemur 15, lateral view **22** ventral spine on trochanter 15, lateral view **23** leg 11, lateral view **24** leg 10, lateral view. Scale bars: 0.05 mm (**20–22**), 0.1 mm (**23, 24**).

Holotype ♂. Body ca 4.0 mm long, ca 0.4 mm wide (in 70% alcohol); colour yellow. Tergites: almost smooth, with relatively long and sparse setae, as in Figs 32–33; T 15 indistinct; posterior margin of TT 1, 3, 5, 8, 10, 12 and 14 slightly sinuate; TT 2, 4, 6 7 9 11 and 13 almost straight; intermediate T slightly elongated, as in Fig. 5. Cephalic plate: width/length ratio 0.8 (width 0.4 mm, length 0.5 mm). Antennae short, reaching the middle of T3, composed of 15+15 short moniliform articles (Fig. 13). Ocelli absent; Tömösváry’s organ very large, oval (Fig. 2). The sides of the labrum with poorly-expressed fringes of bristles; a pair of setae projecting across the labral midpiece present (Figs 14–15). Gnathal edge of mandible with 4 pairs of well-developed teeth and 3–4 rather thick aciculae (Fig. 6). First maxillae: edge with 5–6 plumose bristles and simple setae as well (Figs 28, 36). Second maxillary telopodite with simple and plumose bristles on the tip (two plumose bristles on the left and right parts, respectively) (Fig. 20). Forcipulae: dental margin of coxosternite almost straight, with 3+3 teeth and long setiform porodonts, teeth relatively large, separated from each other by distances less than width at the base of a tooth, median diastema V-shaped; shoulders of coxosternite strongly sloping, as in Figs 1, 4; claw as in Figs 18–19.

**Figures 25–29.**
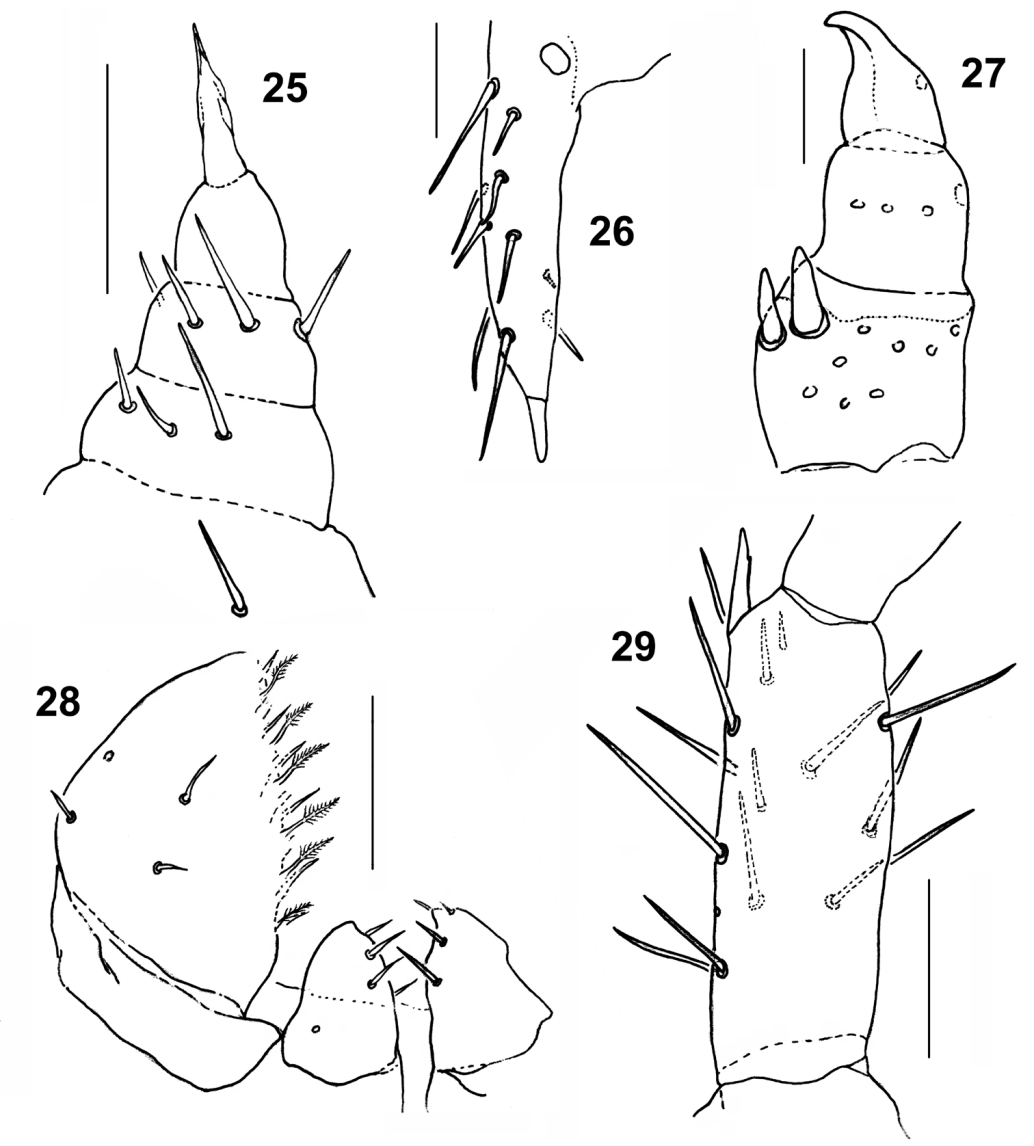
*Shikokuobiusaltaicus* sp. n., male holotype (**25, 26, 28, 29**) and female paratype (**27**). **25** left gonopod, ventral view **26** left mesodistal process on 15 coxa, ventral view **27** left gonopod, ventral view **28** left part of first maxilla **29** distodorsal process on tibia 3, lateral view. Scale bar: 0.05 mm.

Tarsal articulation of legs 1–12 indistinct, tarsi distinctly longer than tibiae. 1–10 tibiae with a distal spinose projection, as in Figs 7–8, 24. 1–14 legs with two accessory spines. 14 and 15 legs not incrassate, with long setae (Figs 11–12). 15 leg: P, F and T relatively short and thick (Fig. 11); C ventrally with a long process (Fig. 26); t and P ventrally with bifurcate spines (Figs 11, 21–22); tarsus 2 with a small distodorsal projection (Fig. 11, shown by arrow). 14–13 legs with the same, but less strongly expressed distodorsal projections. Accessory spines on 15 leg small, poorly-developed (Figs 16–17). At least 13–15 legs dorsally with trace of a broken spine or process (Figs 9, 11–12). A single coxal pore on each of 12–15 legs small and rounded (Fig. 3). Gonopods 4-segmented including terminal filament; 1^st^ segment with three, 2^nd^ segment with four long setae on the external face (Fig. 25).

Paratype ♂. Length 4.0 mm, width 0.4 mm. All other characters as in holotype, but coxal process on leg 15 broken off on both legs.

Non-type material ♂. Length 4.9 mm, width 0.5 mm. All other characters as in holotype (Figs 39–40, 42), but antennae with 17+17 articles, first maxillae with at least six plumose bristles (Fig. 37); second maxillae with four plumose bristles; 14 C ventrally with a tiny denticle, as in Fig. 38; 15 P with a bifurcate spine at distodorsal end (Figs 41, 43); 15 leg with a single well-developed accessory spine; 3^rd^ gonopodal segment with two long setae on the external face.

Paratype ♀♀. All characters as in ♂♂. The number of antennomeres in females unknown: one ♀ with antennae completely broken off, while another ♀ with damaged antennae, having 12+7 antennal articles. Coxal pores as in holotype, formula 1,1,1,2 (Figs 34–35). The number of accessory spines unknown: both females had no 15 leg-pairs. Gonopods without setae on internal face, with 2+2 conical spurs and simple claw (Fig. 27). All segments of gonopods with long setae (broken off as in Fig. 27): 1^st^ segment with eight setae, 2^nd^ with four ones, while 3^rd^ with a single seta on the external face of gonopod.

##### Habitats.

The new species was collected in the lowland Altais in small-leaved and mixed taiga forests at 450 to 920 m a.s.l. (Fig. 45), mainly in soil samples, frequently in deep layers down to 40 cm.

##### Remarks.

The new species belongs to the genus *Shikokuobius* Shinohara, 1982 that shows the following synapomorphies: antenna with up to 18 articles, 3+3 coxosternal teeth; spiracles on leg-bearing segments 3, 5, 8, 10, 12 and 14; coxal pores on 12–15 legs; 15 C with a prominent, acute, mesodistal process; 15 t and 15 P with spines, ventrally bifurcated at their tips; at least 15 P with a bifurcate spine at distodorsal end (as some specimens with spines apparently broken off, so these are not visible).

*S.altaicus* sp. n. is similar to *S.japonicus* (Murakami, 1967), so far the single species in the genus *Shikokuobius*, with the above characters. The main differences between them are given in Table 1. Besides this, the new species differs from *S.japonicus* by: (1) a small distodorsal process on tarsus 2 of legs 13–15 (absent from *S.japonicus*); (2) the number of coxal pores (1,1,1,1(2) in *S.altaicus* sp. n. vs. 2(1),2,2,2 sensu Murakami 1967 and 2,2,2,2(3) sensu Shinohara 1982 in *S.japonicus*).

Finally, *S.altaicus* sp. n. is also rather similar to *Ghilaroviellavaliachmedovi* Zalesskaja, 1975, from the Tajikistan in showing the same body length, simple and plumose bristles on the second maxillae; the number of antennomeres, 1–2 coxal pores, 2+2 spurs and a simple ♀ gonopodal claw. However, *S.altaicus* sp. n. is well-distinguished from the latter species by: (1) 3+3 coxosternal teeth (vs. 2+2 in *G.valiachmedovi*); (2) coxal process well-developed only on leg 15 (vs. on legs 14 and 15 in *G.valiachmedovi*) and (3) the absence of small warts at the base of the ♀ gonopodal claw (vs. 2 small warts in *G.valiachmedovi*).

**Figures 30–35.**
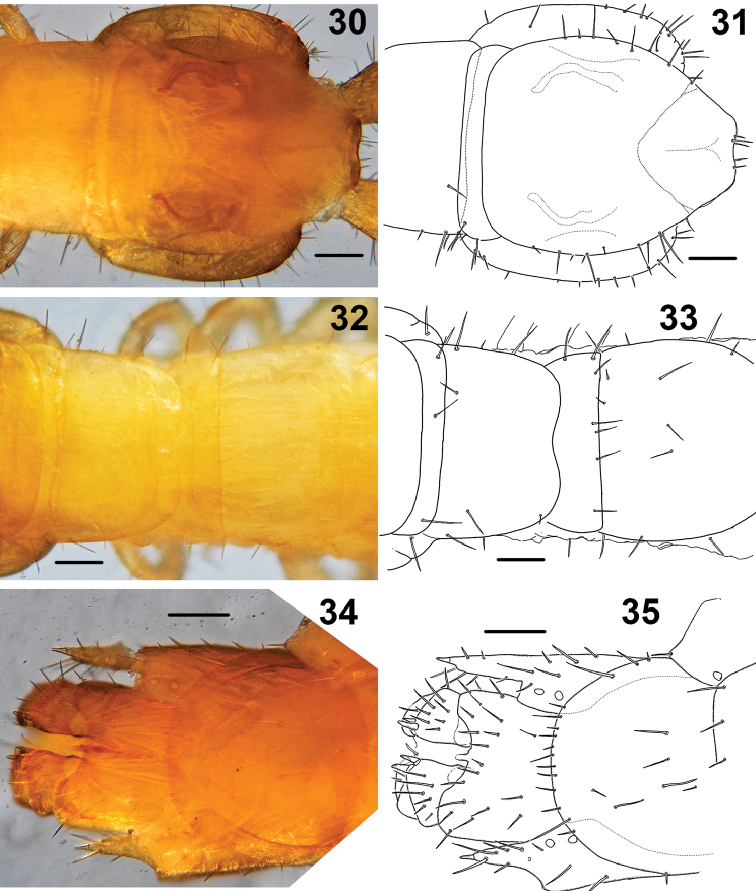
*Shikokuobiusaltaicus* sp. n., female paratype. **30, 31** front body part, dorsal view **32, 33** forcipular and 1–3 leg-bearing segments, dorsal view **34, 35** rear body part, ventral view. Scale bar: 0.1 mm.

**Figures 36–43.**
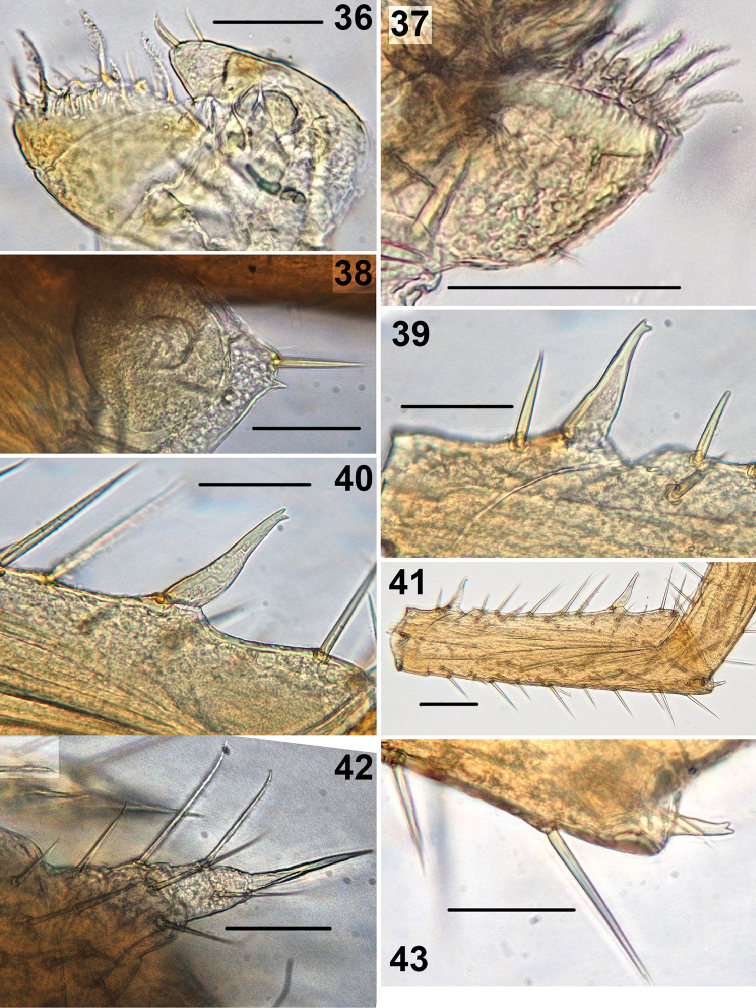
*Shikokuobiusaltaicus* sp. n., male paratype (**36**) and not-type male (**37–43**). **36** left part of first maxilla, ventral view **37** right part of first maxilla, ventral view **38** distal part of coxa 15, ventral view **39** spine on trochanter 15, lateral view **40** spine on prefemur 15, lateral view **41** trochanter and prefemur 15, lateral view **42** right gonopod, ventral view **43** distodorsal spine on prefemur 15, lateral view. Scale bars: 0.05 mm (**36–40, 42, 43**), 0.1 mm (**41**).

**Figure 44.**
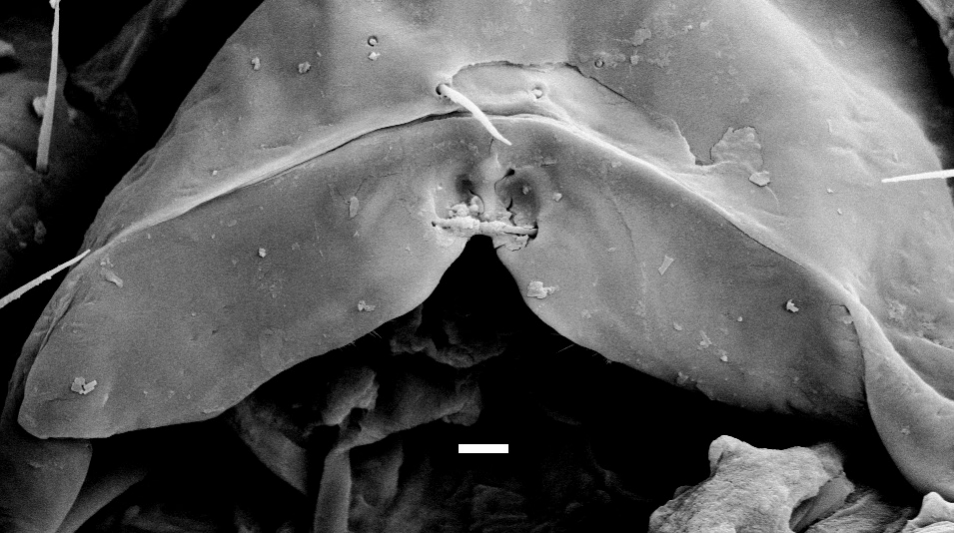
*Shikokuobiusjaponicus* (Murakami, 1967), DNA voucher specimen of Edgecombe and Giribet (2003, 2004), labrum, ventral view. Scale bar: 0.01 mm (courtesy of G.D. Edgecombe).

**Figure 45.**
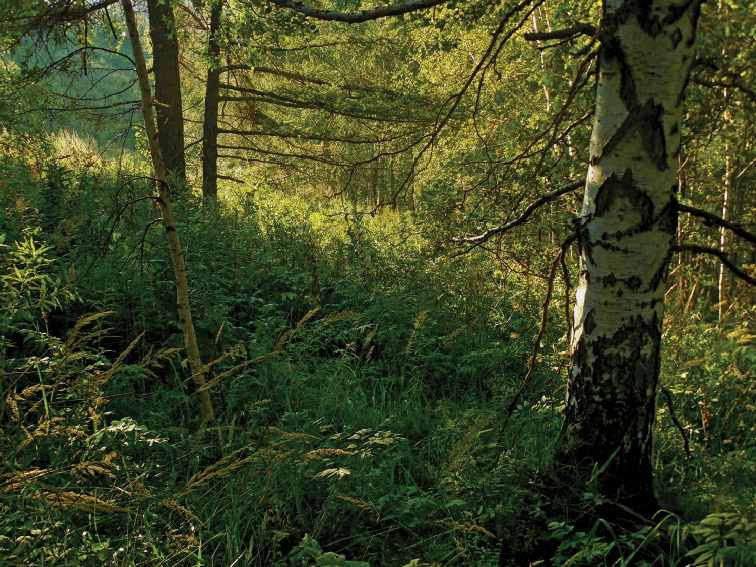
Habitat of *Shikokuobiusaltaicus* sp. n. (♀, PSU-612) in the Tigirek State Nature Reserve (courtesy of T.M. Krugova).

**Figure 46.**
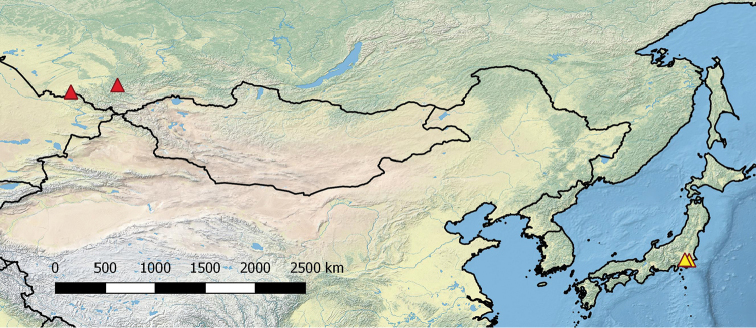
Distribution of *Shikokuobius* species: *altaicus* sp. n. (red triangle) and *japonicus* (Murakami, 1967) (yellow triangle).

**Table 1.** The main differences between *S.japonicus* (Murakami, 1967) and *S.altaicus* sp. n.

	***S.japonicus* (Murakami, 1967)**		***S.altaicus* sp. n.**
	***sensu*Murakami 1967**	***sensu*Shinohara 1982**	
front body part	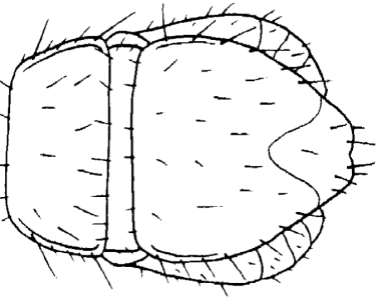	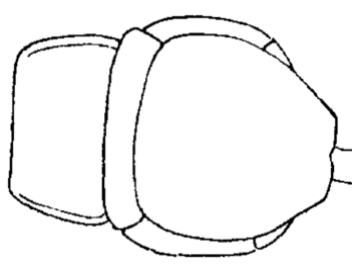	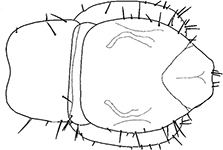 cephalic plate slightly elongate, width/length ratio 0.8; posterior margin of T1 slightly sinuate
	cephalic plate equal in width and length; posterior margin of T1 straight		
number of antennomeres	18	up to 18	15*
labrum	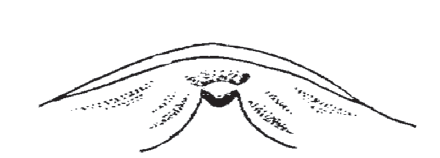 sides are smooth; pair of setae projecting across the labral midpiece absent**	no data	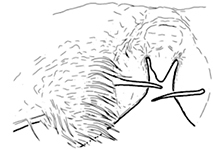 sides with poorly-expressed fringes of bristles; pair of setae projecting across the labral midpiece present
forcipular coxosternite	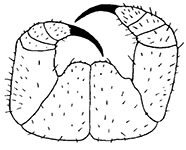	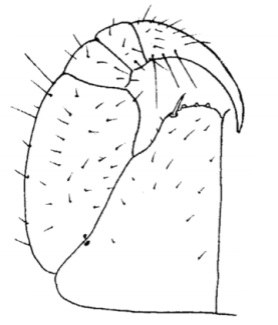	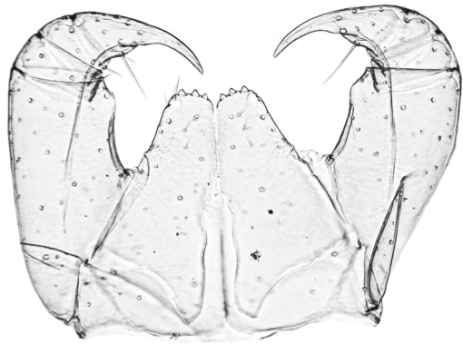 narrower, width/length ratio 1.2–1.3:1
	approximately broad, width/length ratio 1.6–1.8:1		
dental margin of forcipulae coxosternite	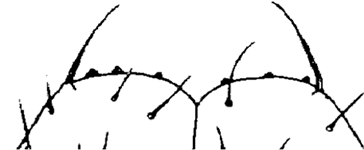	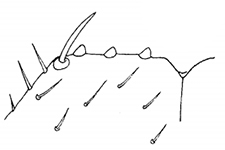	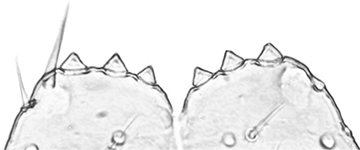 teeth relatively large, separated from each other by distances less than width at the base of a tooth
	teeth very small, separated from each other by distances more than width at the base of a tooth		
leg 15	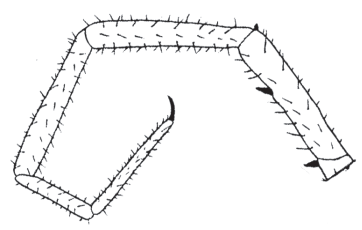	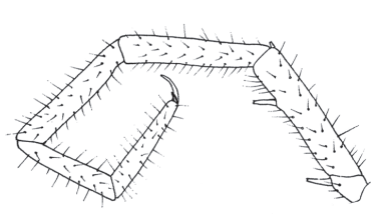	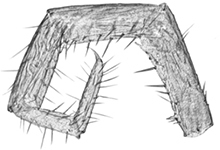 P, F & T shorter and thicker
	P, F & T elongate and thin		

* antennae in a single male (non-type material) with 17+17 articles

** the apparent absence of a pair of setae projecting across the labral midpiece is likely due to them not being noticed by the author of the original description, but sensu G.D. Edgecombe (previously unpublished SEM image of the DNA voucher specimen of Edgecombe and Giribet (2003, 2004) as in Fig. 44), this pair of setae present.

## Supplementary Material

XML Treatment for Shikokuobius altaicus

## References

[B1] Attems C (1938) Die von Dr. C. Dawydoff in Französisch Indochina gesammelten Myriopoden. Mémoires du Muséum d’Histoire Naturelle (Paris) NS 6(2): 187–353.

[B2] Bonato L Edgecombe GD Lewis JGE Minelli A Pereira LA Shelley RM Zapparoli M (2010) A common terminology for the external anatomy of centipedes (Chilopoda). ZooKeys 69: 17–51. 10.3897/zookeys.69.737 PMC3088443 21594038

[B3] Edgecombe GD Giribet G (2003) Relationships of Henicopidae (Chilopoda: Lithobiomorpha): New molecular data, classification and biogeography. African Invertebrates 44: 13–38.

[B4] Edgecombe GD Giribet G (2004) Molecular phylogeny of Australasian anopsobiine centipedes (Chilopoda: Lithobiomorpha). Invertebrate Systematics 18: 235–49. 10.1071/IS03033

[B5] Farzalieva GSh Zalesskaja NT Edgecombe GD (2004) A new genus and species of lithobiomorph centipede (Chilopoda: Lithobiomorpha: Anopsobiidae) from eastern Kazakhstan. Arthropoda Selecta 13(4): 219–224.

[B6] Krasheninnikov AB (2011) Mounting technique of entomological preparations in sandarac medium. Eurasian entomological journal 10(3): 278–279.

[B7] Murakami Y (1967) Postembryonic development of the common Myriapoda of Japan XXIV. A new species of the family Henicopidae. Zoological Magazine, Tokyo, 76: 7–12.

[B8] Shear WA (2018) The centipede family Anopsobiidae new to North America, with the description of a new genus and species and notes on the Henicopidae of North America and the Anopsobiidae of the Northern Hemisphere (Chilopoda, Lithobiomorpha). Zootaxa 4422(2): 259–283. 10.11646/zootaxa.4422.2.6 30313504

[B9] Shinohara K (1982) A new genus of centipede of the subfamily Anopsobiinae (Henicopidae, Chilopoda). Proceedings of the Japanese Society for Systematic Zoology 24: 41–46.

[B10] Silvestri F (1909) Descrizioni preliminari di vari artropodi specialmente d’America. Rendiconti della R. Accademia dei Lincei. Classe di Scienze Fisiche Matematiche e Naturali 18: 267–271.

[B11] Zalesskaja NT (1975) [New genera and species of Chilopoda (Lithobiomorpha) from Central Asia and Far East]. Zoologicheskii Zhurnal 54(9): 1316–1325. [in Russian]

[B12] Zapparoli M Edgecombe GD (2011) Order Lithobiomorpha. In: Minelli A (Ed.) Treatise on Zoology – Anatomy, Taxonomy, Biology – The Myriapoda, Volume 1.Brill, Leiden – Boston, 371–389.

